# New regression formula to estimate the prenatal crown formation time of human deciduous central incisors derived from a Roman Imperial sample (Velia, Salerno, Italy, I-II cent. CE)

**DOI:** 10.1371/journal.pone.0180104

**Published:** 2017-07-12

**Authors:** Alessia Nava, Luca Bondioli, Alfredo Coppa, Christopher Dean, Paola Francesca Rossi, Clément Zanolli

**Affiliations:** 1 Dipartimento di Biologia Ambientale, Università di Roma ‘La Sapienza’, Rome, Italy; 2 Museo delle Civiltà. Museo Nazionale Preistorico Etnografico ‘Luigi Pigorini’, Section of Bioarchaeology, Rome, Italy; 3 Department of Cell and Developmental Biology, University College London, London, United Kingdom; 4 Laboratoire AMIS, UMR 5288, Université Toulouse III, Toulouse, France; Monash University, AUSTRALIA

## Abstract

The characterization and quantification of human dental enamel microstructure, in both permanent and deciduous teeth, allows us to document crucial growth parameters and to identify stressful events, thus contributing to the reconstruction of the past life history of an individual. Most studies to date have focused on the more accessible post-natal portion of the deciduous dental enamel, even though the analysis of prenatal enamel is pivotal in understanding fetal growth, and reveals information about the mother’s health status during pregnancy. This contribution reports new data describing the prenatal enamel development of 18 central deciduous incisors from the Imperial Roman necropolis of Velia (I-II century CE, Salerno, Italy). Histomorphometrical analysis was performed to collect data on prenatal crown formation times, daily secretion rates and enamel extension rates. Results for the Velia sample allowed us to derive a new regression formula, using a robust statistical approach, that describes the average rates of deciduous enamel formation. This can now be used as a reference for pre-industrial populations. The same regression formula, even when daily incremental markings are difficult to visualize, may provide a clue to predicting the proportion of infants born full term and pre-term in an archaeological series.

## Introduction

Human teeth are (paleo)biological archives capable of permanently recording an individual's developmental growth history. A number of classic papers have described the incremental nature of enamel growth and also suggested the likely circadian nature of enamel short period-markers and the regular longer-period rhythm of incremental markings associated with surface enamel perikymata [1–8]. The first use of these enamel incremental structures in clinical dentistry to put a time scale to tooth crown formation was by Schour and Massler [9], Kajiyama [10], and Takiguchi [11]. However, Schour [12] was the first to fully describe the neonatal line in deciduous teeth as a marker of birth and Boyde [13] was the first to use enamel incremental markings to establish an age at death in archaeological material. The history of these early studies has been fully reviewed by Boyde [14], Dean [15], FitzGerald [16], Smith [17], and most recently and comprehensively by Hillson [18]. In addition, the evidence for the daily incremental nature of enamel cross striations has been strengthened with new evidence that clock-genes operate during amelogenesis [19–22].

Dental enamel does not undergo remodeling and preserves its original structure unchanged through life [23]. Therefore, the histomorphometrical characterization of preserved mineralized enamel growth increments enables the retrieval of information about the following: the Crown Formation Time (CFT [24–28]); the Daily enamel Secretion Rate (DSR, the speed at which the ameloblast–the enamel forming cells–move towards the outer surface of the tooth [24–30]); the Enamel Extension Rate (EER, that is, according to Shellis [31], the rate of differentiation of secretory ameloblasts, or the speed at which ameloblasts into the secretory front are recruited along the enamel-dentine junction–EDJ–between the dentine horn in the cusp towards the enamel cervix [28,31–33]); the health status and, in individuals with still forming crowns, the age at death [14,29,34–40]. The rhythmical growth of enamel is expressed in humans at two different levels: a circadian scale that produces the cross-striations and a longer period scale (near- weekly in humans) that give rise to the regular Retzius lines (rev. in [41]). Physiological stresses exceeding a certain threshold leave their permanent marks in the corresponding position of the secretory ameloblast front, producing Accentuated Lines (AL or Wilson bands [9, 41, 42]) that are superimposed onto the regular physiological growth markings. The birth event is recorded in the forming enamel of individuals surviving the perinatal stage, and leaves an Accentuated Line, known as Neonatal Line (NL, rev. in [43, 44]). The NL separates the enamel or dentine formed prenatally from that growing after birth and constitutes a reference point (age zero) with which to calibrate a daily time scale of the forming dental enamel. In individuals that survived at least 10–15 days after birth [38, 43], but maybe even less [45], the NL is thus present in all the crowns that started to form *in utero*, i.e. all the deciduous crowns and usually the protoconid of the first permanent mandibular molar.

However, studies that have used enamel incremental growth specifically to analyze deciduous crown formation times are fewer than those on permanent teeth. Dean [46] and Macchiarelli [28] described a faster overall trajectory of occlusal enamel growth in deciduous molars than in permanent modern human molars and Mahoney [26], Birch [47], Birch and Dean [27, 48] used daily enamel increments in deciduous teeth to estimate crown formation times.

According to Hillson [18], "Brown striae [Retzius lines] are less prominent in prenatal enamel matrix then they are in the post-natal matrix, but cross striations can be counted in places". This general homogeneity can be attributed to the protected and buffered environment in which the tissue develops [49, 50].

The estimates of DSR and EER on prenatal enamel are mostly available from modern reference collections of exfoliated/extracted deciduous teeth [25–28, 31, 32, 47, 48]. Studies on deciduous DSR report a topographical variation of the rates in different part of the tooth crown: the speed at which the ameloblasts move and secrete enamel matrix is lowest near the EDJ, but accelerates toward the outer enamel surface and slows down toward the end of enamel formation at the cervix [48]. Mahoney [26], on a Medieval and contemporary tooth series, observed that: "the prenatal enamel growth trajectory for deciduous incisors differed relative to the other tooth types. Incisors combined rapid growth with initiation early on in the second trimester [of pregnancy] to produce a greater proportion of their crown before birth than any other tooth type". According to Mahoney [26], the DSR in the cuspal enamel of maxillary deciduous central incisor ranges from a mean of 3.59 μm day^-1^(sd = 0.39) close to the EDJ, to a mean of 5.15 μm day^-1^ (sd = 0.19) moving towards the outer enamel.

Linear regression equations were derived by Mahoney [25] for deciduous incisors with unworn enamel and those where the occlusal enamel is worn but the EDJ remains intact (unworn incisors: CFT = 122.980 +11.357 x [area of tooth crown section in mm^2^]; worn incisors: CFT = -236.268+43.751 x [length of EDJ in mm]). Birch and Dean [27] used cumulative daily cross-striation counts made along defined prism paths recorded at every 100 μm, or final 50 μm of enamel thickness, from the EDJ to the enamel surface to estimate the DSR in a sample of modern deciduous teeth. Linear regression equations were also generated from these cumulative cross-striation counts by plotting them against linear enamel thickness along a given prism path. Therefore, the previous contributions provide an expeditious way to predict the CFT of the deciduous dentition.

The present paper reports new data describing prenatal crown formation times (pCFT), DSR and EER of central deciduous incisors from the Imperial Roman necropolis of Velia (I-II century CE, Salerno, Italy) [51, 52]. In this study we concentrate on prenatal enamel formation in deciduous central incisors because a greater amount of enamel forms prenatally in deciduous central incisors than in any other tooth [26]. Since the dental series used by Birch and Dean [27] was composed of a contemporary clinically extracted sample of deciduous teeth, we expect there to be differences between this present pre-industrial sample and the modern one. These differences are likely to add to the inter-population variability we expect to observe among modern human populations today but they are also likely to reflect the additional health-related constraints experienced by the mother and fetus during development in this archaeological population. Indeed, new data from pre-industrial populations are required to build a more consistent and appropriate reference dataset for the analysis of archaeological and even of paleoanthropological dental specimens. Moreover, the Velia dataset has been used to derive a new regression formula to estimate human deciduous central incisors prenatal crown formation time as reference for pre-industrial populations. Given the large sample available, it was the specific aim of this paper to contribute towards a better understanding of the temporal events and processes underlying prenatal and perinatal enamel development in ancient populations. Potentially, these new data may in the future make an important contribution to studies that aim to estimate the proportion of infants in archaeological populations born pre-term or full-term [45] and to estimates of age at death of infants when daily incremental markings are difficult to visualize [38, 53].

## Materials and methods

Histological analysis was conducted on the deciduous dentition of infants from the large Imperial Roman necropolis at the ancient port of Velia (I-II centuries CE, Campania, Southern Italy) [54]. The ancient city of Velia was founded as a Greek colony (originally named Elea) around 540 BCE, on the west coast of Italy, south of Salerno. The city, under Roman control since the late third century CE, functioned as a trading centre and port. During the Middle Ages Velia came under episcopal control and, after a period of fast decline, was completely destroyed between VIII and IX centuries CE. The skeletal collection of Velia is kept at the Museo delle Civiltà - Museo Nazionale Preistorico Etnografico Luigi Pigorini of Rome to which three authors (AN, LB, and PFR) are affiliated. No permission is needed for these analyses.

Eighteen deciduous central incisors (12 upper and 6 lower) from the necropolis of Velia were histologically sectioned along the central labiolingual plane and prepared as thin sections with a final thickness of about 100 μm, following the method in [55]. Specimens with absence of incisal wear and where there was good visibility of cross-striations in the prenatal enamel were selected for further study. The selected dental sample consists of crowns that were not fully formed at the moment of death from individuals interred in single graves. The morphological (skeletal and dental) age at death estimation (reported in S1 Table), together with the single interment of the bodies, ensures us that all these individuals were born and, following Scheuer and Black [56], died whether perinatally (around the time of birth) or as neonate (first 4 weeks after birth) or infants (birth to the end of the first year). Therefore, the absence of the NL means that the individuals didn’t survive enough to form the birth landmark [38, 43, 45]. In those cases, where the NL was absent, the individuals died soon after birth and the last formed enamel of the crown can be considered as almost coincident with birth and death. Accordingly, in the absence of the NL all the observable enamel can be considered as prenatal (see S1 Fig). Moreover, the series more than likely includes pre-term individuals that contribute to the distribution of age at death and sample structure.

The macroscopic age at death assessments of Velia’s individuals were based on the developmental stages of deciduous and permanent teeth [57, 58] and on the diaphyseal length of long bones [59, 60].

Micrographs of each histological section were prepared at 200x of magnification, by acquiring a series of overlapping pictures under polarized light using a high resolution camera (Leica DFC 295) attached to an optical transmitted light microscope (Laborlux S Leica AG). The photomontages were assembled through the software Microsoft Image Composite Editor (ICE version 2.0.3.0–64 bit).

The time taken to form each crown prior to birth was calculated following the method described in [27, 33, 61]. Segments of various length were traced and measured along single enamel prisms between the EDJ and a biological landmark (AL or physiological line). Starting from the tip of the dentine horn and avoiding the gnarled central cuspal enamel, the line was followed back to the EDJ. Starting from this point, the same procedure was repeated as many times as necessary until reaching the most cervical available point of the crown or the Neonatal Line. For each segment along the prisms the total formation time was determined by direct count of the cross-striations. Two observers performed all counts independently (AN and LB), after a common agreement on the identity of the biological landmarks. The measured prism lengths do not exceed 200 μm (following Birch and Dean [27]) except in a few cases where the long period markers were not clearly discernible in the first 200 μm (S2 Table). Indeed, the correlation of the DSR with the prism length is very low (Pearson's R = 0.18) and it is not significantly different from zero. This low correlation ensures that the different lengths of the prisms did not affect the consistency of the DSR evaluation.

The final cross-striation counts were obtained as the average of the repeated measurements (coefficient of reliability R = 0.96 [62]; t test for repeated measurements t = -0.05, df = 74, p = 0.96). Distances were plotted against cross-striation counts to derive the regression formula for the deciduous central incisors. In order to compensate for the presence of possible outliers, a robust regression method was adopted [63], with the constraint of the intercept equal to zero (no prism length equal to no days of enamel matrix production).

The cross-striation counts of each prism's portion were used to calculate the DSRs. Measurements of the EDJ length between biological markers, and the corresponding number of days taken to form that length, were used to derive the EER as micrometers per day. Measurements along the EDJ were repeated from the tip of the dentine horn until the NL or the end of the crown [32].

In order to better illustrate the changes of the DSR and of the EER along the EDJ of the developing crown, the EDJ length of all teeth has been divided into three segments of different proportion of the total length: a. the cuspal-middle portion that covers the 0–70% of the prenatal EDJ length; b. the middle-neonatal portion that covers the 70–90% of the prenatal EDJ length; c. the neonatal portion that covers the remaining 10% of the prenatal EDJ length. The three segments of unequal length were chosen because the EER in the cuspal region of the central deciduous incisors forms very fast. Consequently, any cross-striation count along a rather short prism length, when close to the tip of the dentine horn, corresponds to a very long fraction of the total EDJ length forming at the same time.

Finally, for one individual (Velia T.237), the topographical distribution of the DSR was calculated by collecting random measurements (N>100) along the prisms and recording the corresponding cross-striations counts between the EDJ and the surface enamel. The corresponding spatial distribution of the DSR was calculated from the raw data using a surface obtained from a Generalized Additive Model fit [64].

The pCFT variation profiles with reference to the EDJ were calculated with a locally weighted polynomial regression fit [65] of the lengths on the EDJ against the prenatal CFTs (data from S2 Table).

All regression analyses and graphs were performed using the R statistical package ver. 3.3.3 [66], together with the robustbase (Basic Robust Statistics package, version 0.92–6 [67]) and the mgcv packages (mgcv package, version 1.8–17 [64]).

## Results

Direct counts of cross-striations were made in all the crowns from the Velia series. Table 1 reports the individual crown formation times of the prenatal portion of the incisors' crowns (pCFT). The mean of the pCFT for the upper central incisors is 125 days (sd = 28.6, n = 12) and 107 days (sd = 19.1, n = 6) for the lower ones. S2 Table reports the individual cross-striations counts, prism lengths and EDJ measurements. The analysis of covariance (ANCOVA) run with the prism length as the dependent variable, the pCFT as the covariate and the dental arch as the factor, shows no significant interaction between the pCFT and the arches (F = 1.415, df = 1, p = 0.24).

**Table 1:** Individual prenatal enamel growth parameters in the Velia series. The predicted values of pCFT from the new regression formula and the residuals from the direct count are also reported.

ID	tooth class and presence of the NL	prenatal CFT (days) direct count	prenatal CFT (days) estimated by the new regression	deviation direct count-new regression	DSR in μm/day (sd; n)	Cuspal- Middle EER in μm/day	EER in μm/day (sd; n)
T98	upper present	179	145	34	3.81 (0.20; 3)	54.0	30.77 (21.25, 3)
T142	lower absent	105	97	8	4.28 (0.24; 3)	58.0	42.70 (21.64;2)
T155 I	upper absent	145	127	18	4.18 (0.60; 4)	37.7	28.33 (8.81;3)
T168 I	upper absent	120	120	0	4.75 (0.37; 3)	55.4	35.53 (17.64; 3)
T197	upper present	120	114	6	4.25 (0.41; 3)	51.9	38.37 (12.20; 3)
T221	lower absent	76	67	9	4.20 (0.29; 3)	53.8	37.43 (14.59;3)
T229	upper present	138	134	4	4.57 (0.29; 3)	54.5	33,10 (18.65; 3)
T237	lower absent	102	93	9	4.14 (0.33; 4)	54.3	41,90 (10.92; 3)
TT243	upper present	100	111	-11	5.18 (0,17; 4)	84.3	46.65 (25.77; 4)
T252	upper absent	164	133	31	3.63 (0.53; 8)	59.1	31.34 (14.37; 7)
T301	lower absent	109	104	5	4.37 (0.33; 5)	41.1	51.08 (6.66; 4)
T312	upper absent	133	130	3	4.54 (0.29; 6)	63.3	42.28 (12.21; 6)
T330	upper absent	108	118	-10	5.04 (0.29; 5)	55.2	41,50 (14.77; 4)
T344	upper absent	114	130	-16	5.31 (0.40; 5)	59.3	45.98 (14.17; 4)
T349	lower absent	135	146	-11	5.01 (0.33; 5)	67.4	41.17 (22.82; 3)
T399	lower present	114	121	-7	4.95 (0.56; 4)	39.7	35.68 (5.56; 4)
T422	upper absent	73	82	-9	5.22 (0.49; 3)	59.6	48.35 (15.91; 2)
T438	upper absent	111	123	-12	4.98 (0.63; 5)	65.4	51.63 (11.24; 4)

Only five teeth showed the presence of a clear NL (S1 Table), because the corresponding individuals survived birth for a sufficient amount of time for enamel to be preserved beyond the NL (S1 Fig). The remaining 13 individuals did not survive long enough to form a NL or were stillborn. The mean pCFT for the survivors (x = 130.0, sd = 30.0, N = 5) is, as expected, higher that the mean of the individuals that died perinatally (x = 114.7, sd = 25.3, n = 13) even if the difference of the means is not statistically significant (Mann Whitney U test with continuity correction, W = 23, p = 0.37). Moreover, we have to consider that the most cervical and outer portions of the forming enamel, composed only of recently secreted immature enamel matrix, has been lost in the post-depositional time. As noted by Boyde [68] cited by [37], “the amount of tissue likely to be lost from the rotting of immature enamel probably will not amount to more than a week or two’s worth of growth”. Indeed, [38, 45, 53] have demonstrated that this is likely to be the case.

S1 Table also reports the estimated pCFT for the Velia individuals as derived from the regression equations for the central incisors proposed by Birch and Dean [27] and from the regression of the total EDJ length in reference to the CFT, as proposed by Mahoney [25]. In Mahoney [25] the formulae estimate total crown formation from the total enamel area or from the total EDJ length, combining the pre- and post-natal portion of the crown, and are therefore not fully appropriate for estimating the pCFT. The difference between the direct counts and the estimated pCFT from the regression formula in [27] is constantly positive with the mean of 43 days and a standard deviation of 17.06 (range = 9 to 69). The major source of discrepancy is the presence, in the Birch and Dean [27] formula for the central incisor, of an intercept constant of 6.73 days. In their total crown formation time estimates (the sum of both prenatal and postnatal enamel formation times) Birch and Dean [27] summed the estimates of prism formation times made along several prism lengths that together span the whole of enamel formation from the dentine horn in the cusp along the EDJ to the cervix. The value of each individual time estimate includes the same intercept constant (6.73) which, therefore, cumulates and increases the crown formation time by a fixed value for each partial estimate of the total prism length formed during crown formation. Indeed, with this approach, the more segments that are measured, the larger is the discrepancy with the direct account. Therefore, a new regression formula, targeted for the prenatal enamel of the deciduous central incisors, has been derived from the Velia sample that takes count of the effects of including an intercept constant. In order to overcome the effect of the intercept value, the regression has been forced to pass through the origin (no enamel matrix secretion = no formation time).

**Fig 1:**
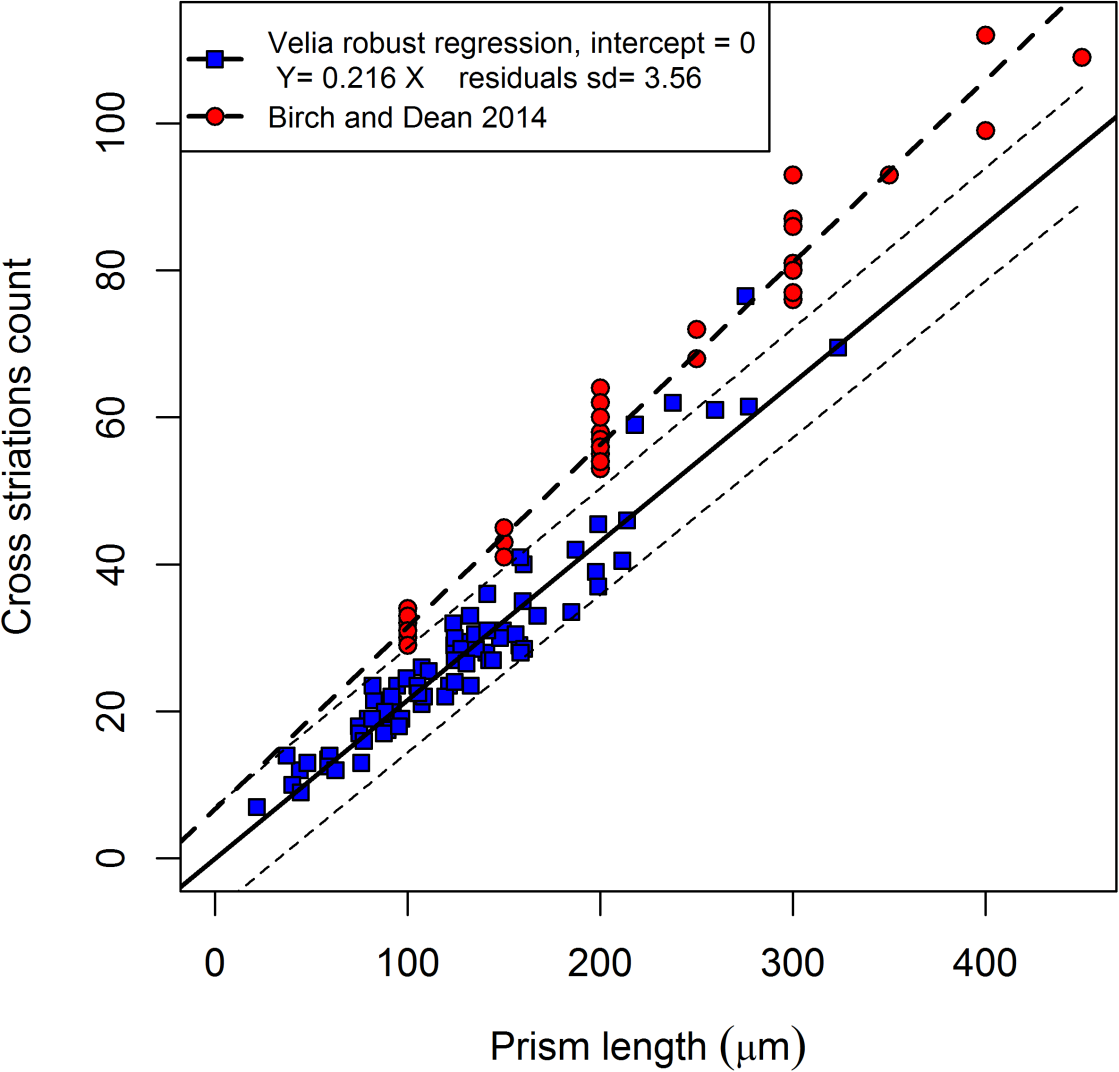
Regression of the Velia cross-striations count against the prism length. The Birch and Dean [27] regression for the central deciduous incisors is also shown.

Fig 1 illustrates the new regression, compared with the published data in Birch and Dean [27] and the corresponding regression. The 95% confidence interval for the predicted values is also shown in the same figure. The robust regression formula for the Velia sample, forced to have intercept equal to zero, is Y = 0.216 X (X = prism length in μm; Y = cross striations count), with the lower 95% boundary Y = 0.208 X and the upper 95% one Y = 0.238 X. With this approach, the inverse of the angular coefficient of the formula represents the estimated overall DSR and is equal to 4.64 μm day^-1^, a value higher than the reported mean DSR of 3.43 μm day^-1^ by Birch and Dean [27], but closer to the Mahoney [26] figures (DSR = 4.25 μm day^-1^). The adjusted R^2^ is 0.986, but the real significance of this parameter is weakened by forcing the intercept to be zero. A polynomial regression fit was attempted using different powers, but the best fit obtained with the cubic do not better explain the total variability (adjusted R^2^ = 0.97) and, consequently, the sum of squared residuals tends to increase in reference to the linear approach.

The fifth column in Table 1 reports the residuals of the new regression formula. The absolute difference between the direct count and the estimated pCFT from the regression has a mean of 11 days and a standard deviation of 8.74 (range = -34 to 16). The mean error of the estimate is 9.8% for the upper dentition, 7.9% for the lower, 8.7% for the survivors (showing the NL), and 9.2% for the non survivors (without the NL). The analysis of the residuals shows that lower mean individual DSR values correspond to the underestimate of the pCFT, and vice versa.

Table 1 reports the individual DSR and EER as calculated by moving along the EDJ from the dentine horn to the cervix for the 18 teeth. The number of measurements along the EDJ in the crown varies from a minimum of 3 segments to a maximum of 8. The mean DSR for all the segments measured in the crown is 4.57 μm day^-1^ (sd = 0.62, n = 76) which differs by 0.7 μm day^-1^ from the robust regression estimate. The mean EER for the whole crown is 40.07 μm day^-1^ (sd = 14.79, n = 65).

**Fig 2:**
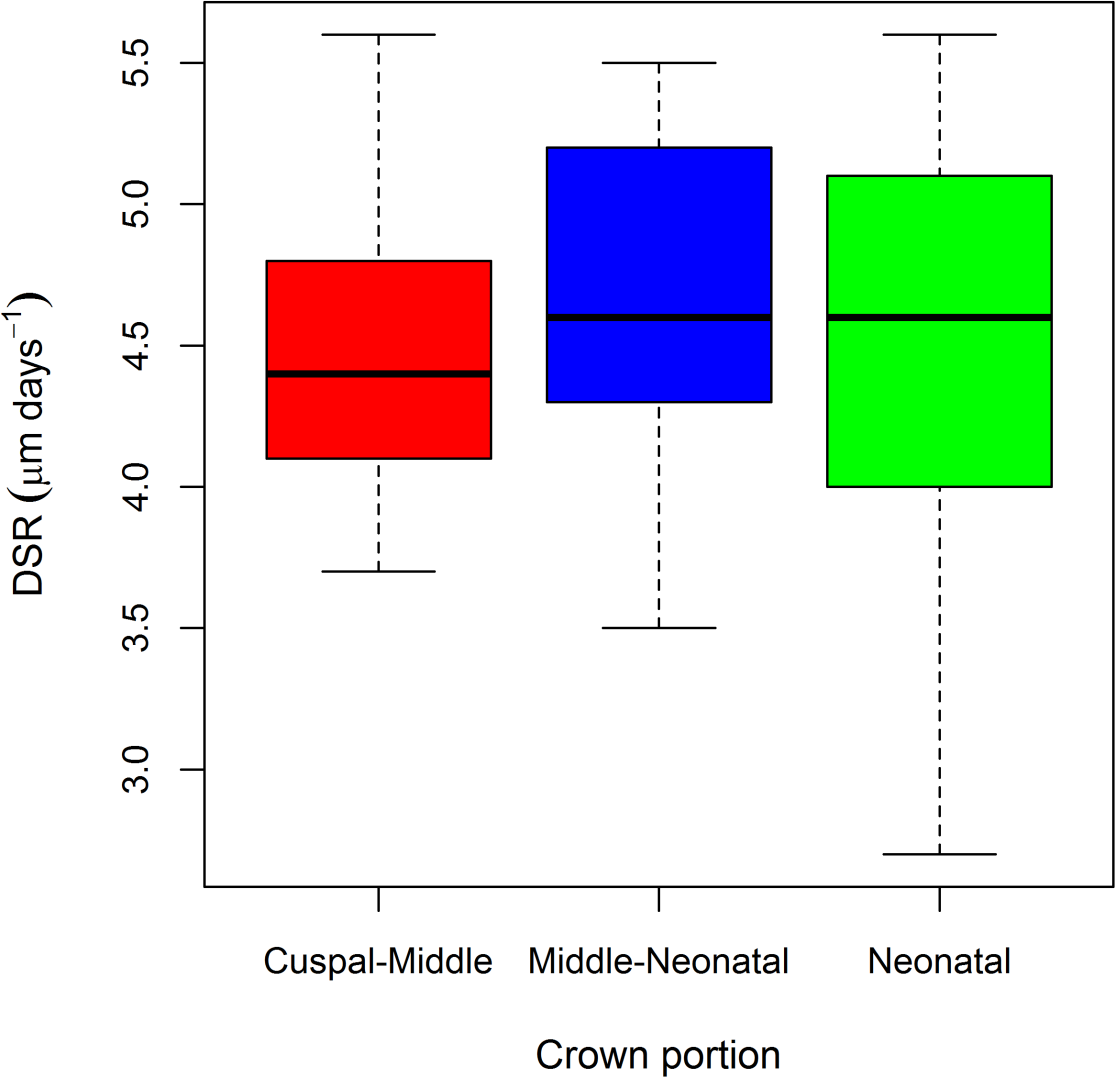
Boxplot of the DSR variation along the EDJ in the Velia sample. The boxplot shows the median, the range (lower and upper whisker), the first quartile (lower hinge) and the third quartile (upper hinge).

Fig 2 reports the boxplots of the DSR distribution along the different portions of the crown. As noted by Birch and Dean [27] the DSR does not change substantially moving from the cuspal to cervical region along the EDJ (comparison of the DSR among regions: Kruskal-Wallis test, chi-squared = 0.92863, df = 2, p-value = 0.6286). However, an increase of the variability moving towards the most cervical forming enamel is noticeable, even if not statistically significant (Levene's test for homogeneity of variance: F = 0.824, df = 2, p-value = 0.434). This suggests that, even for measurements along prism paths that fall beyond the prenatal enamel formation period, the new regression equation (Y = 0.216 X; X = prism length in μm; Y = cross striations count) would in addition more accurately predict cumulative enamel formation times for deciduous incisors than the original regression formula presented in Birch and Dean [27] because the intercept is forced through the origin (see above).

**Fig 3:**
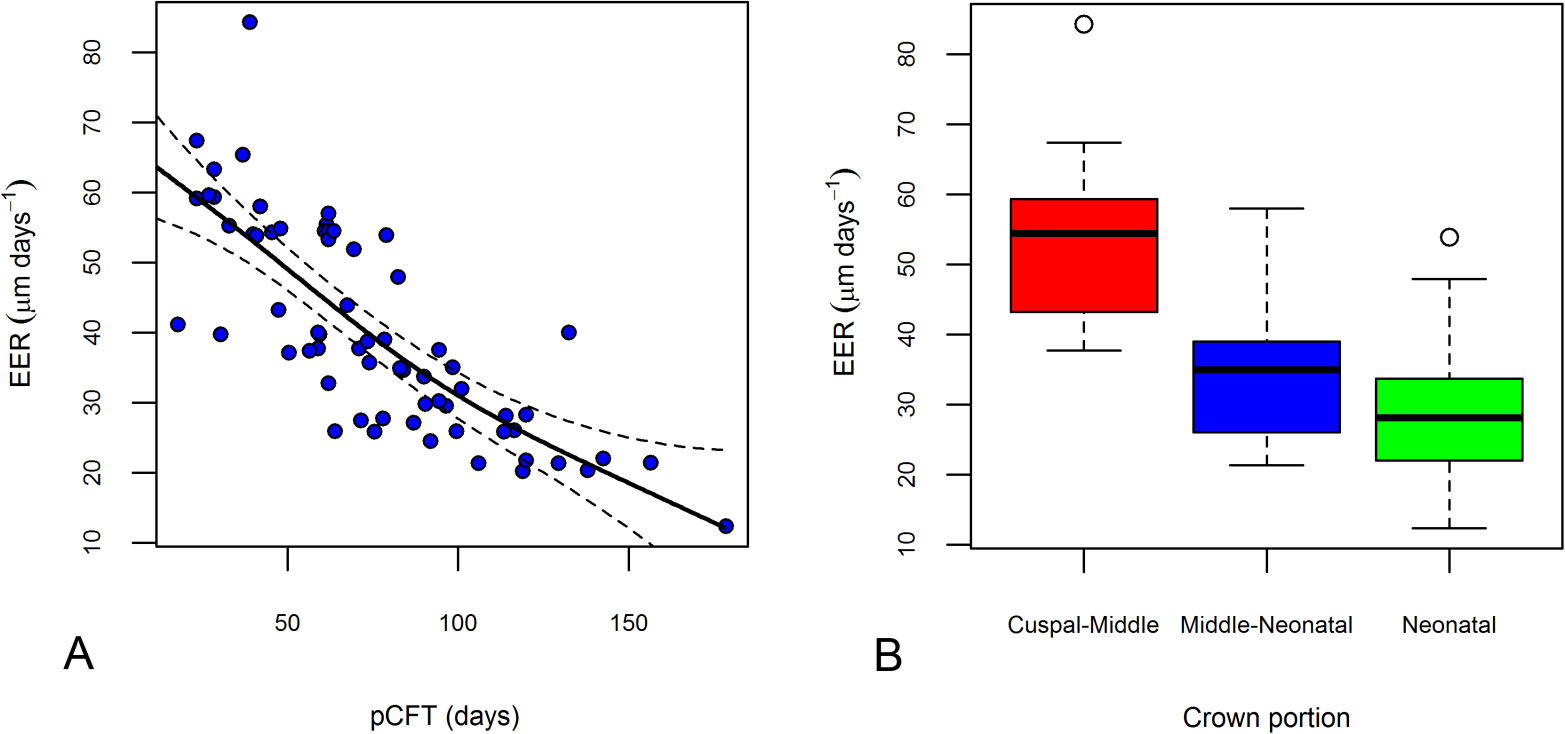
Variation of the EER along the EDJ in the Velia series. (A) Scatterplot of the pCFT against EER. (B) Boxplot of the EER variation along the EDJ. The boxplot shows the median, the range (lower and upper whisker), the first quartile (lower hinge), and the third quartile (upper hinge).

Enamel Extension Rate decelerates sharply through time (i.e. along the EDJ towards the cervix, the correlation between pCFT and EER is negative and significantly different from zero, Pearson's R = -0.781, t = -9.94, df = 63, p-value < 0.001). Fig 3A illustrates the scatter plot of pCFT against EER. The best estimate of this trend in deceleration is given by a Generalized Additive Model [65] predicting the EER from the pCFT with a thin plate spline smooth term for the pCFT. The explained deviance equals 63.2% of the total. Given the non-linear nature of the relationship between the EER and the growing crown, the DSR is only weakly correlated with the EER (Pearson's R = 0.25, t = 2.08, df = 63, p = 0.041).

Fig 3B reports the boxplots of the EER distribution along the previously defined crown segments, as in Fig 2. EER changes substantially (Kruskal-Wallis test: chi-squared = 31.591, df = 2, p-value < 0.001) along the EDJ. Conversely, the variability moving towards the most cervical forming enamel is stable and not statistically significant (Levene's Test for Homogeneity of Variance: F = 0.368, df = 2, p-value = 0.694).

**Fig 4:**
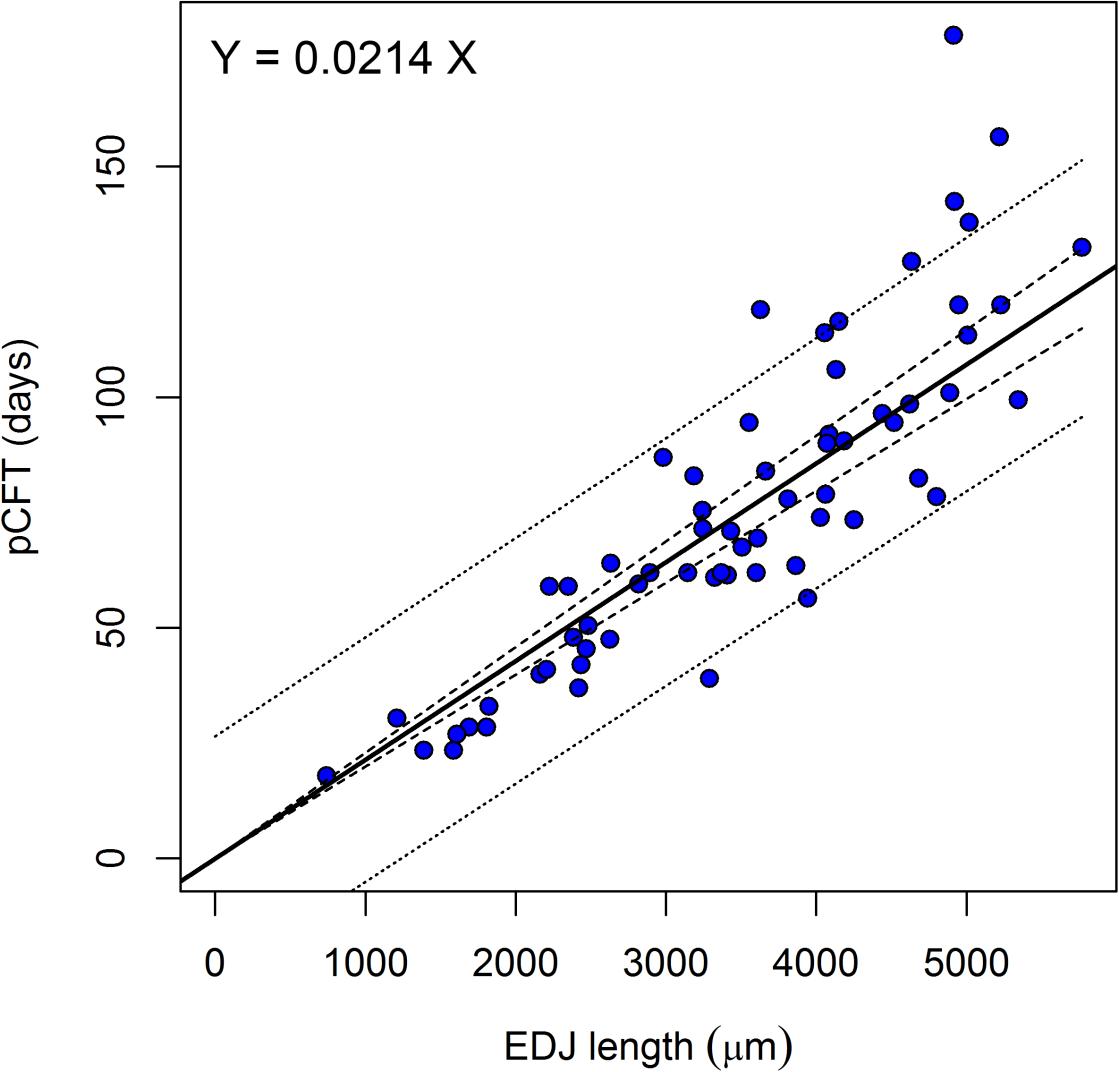
Scatterplot and regression line of the EDJ lengths against the pCFT in the Velia series. The 95% interval of the prediction (dotted lines) is shown together with the 95% confidence interval of the regression (dashed lines).

Fig 4 shows the regression of the pCFT on the EDJ length. The length of the EDJ is positively correlated with the pCFT (R = 0.87, t = 13.87, df = 63, p<0.01). The robust regression formula, forced again to have the intercept equal to zero, is Y = 0.0214 X (X = EDJ length in μm; Y = cross striations count), with the lower 95% boundary Y = 0.02 X and the upper 95% one Y = 0.029 X. The adjusted R^2^ is 0.9649.

Because the EER does not vary linearly along the EDJ, a polynomial regression fit was attempted, but the quadratic terms are not statistically significant and do not better explain the total variability (adjusted R^2^ = 0.81).

**Fig 5:**
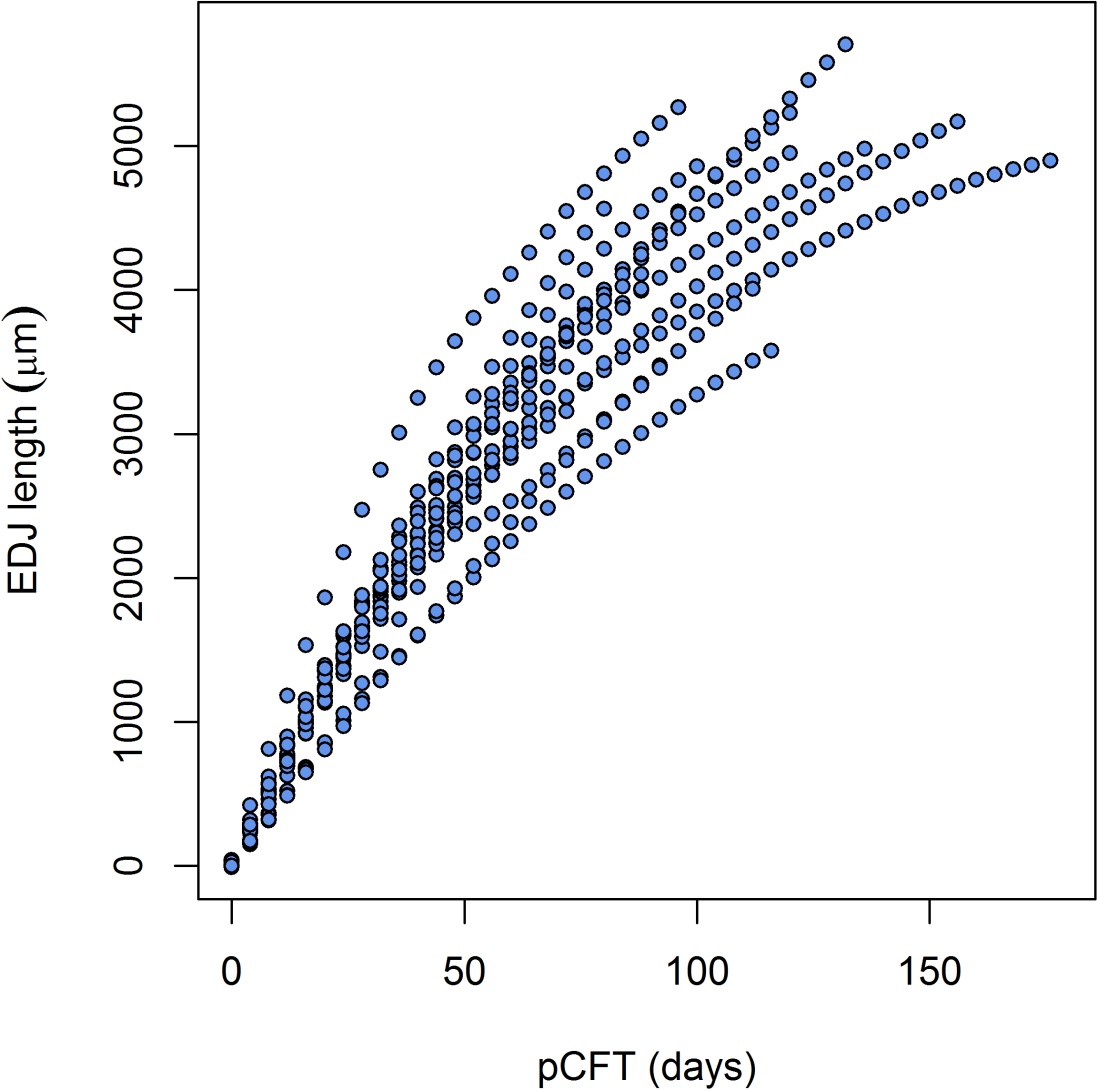
Individual pCFT variation with reference to the EDJ length in the Velia series. Each profile was calculated with a locally weighted polynomial regression fit [65]. See methods section for details.

Fig 5 shows the increase in EDJ length during the time of formation of the crown for all the 18 individuals from Velia. Despite a large inter-individual variability, the general trend shows the clear deceleration in the recruitment of new secretory ameloblasts along the EDJ.

**Fig 6:**
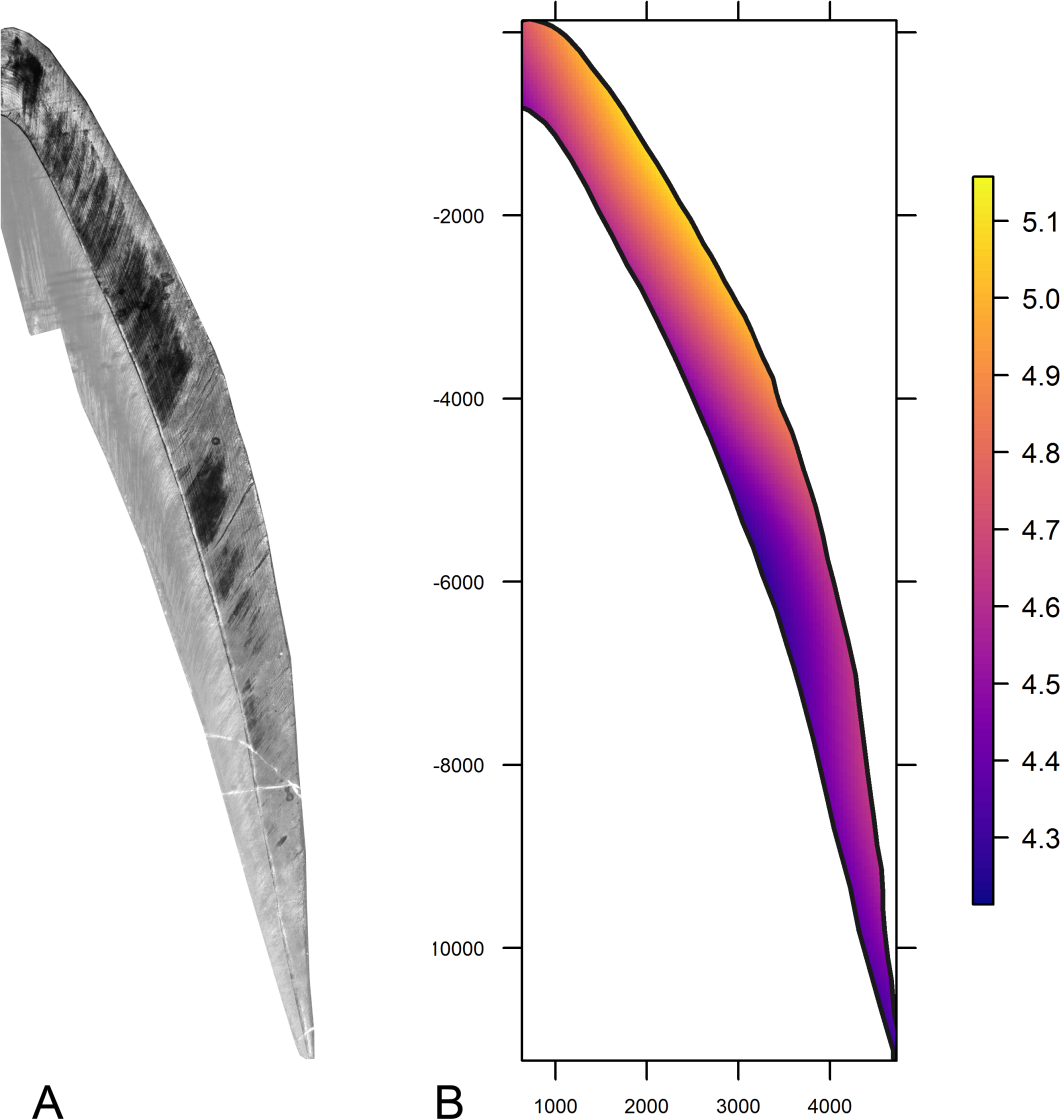
Map of the DSR values across the prenatal portion of the crown of the T.237 individual. (A) Histological section of the buccal aspect of the T.237 central incisor. (B) Topographic distribution of the DSR values on the prenatal portion of the buccal aspect of the same tooth.

Fig 6A reports the histological section of the individual T.237 used to illustrates the topographic distribution of the DSR values on the prenatal portion of the buccal aspect of the lower central incisor. The map (Fig 6B) confirms the general pattern of an almost stable rate of enamel matrix secretion close to the EDJ along the whole of its length with only limited reduction in secretion rate towards the most cervical portion. Conversely, the DSR accelerates across a gradient from the EDJ toward the outer surface of the crown, but still with rates increasing by a micrometer or less per day across the whole thickness of enamel (something that contrasts markedly with permanent enamel [69]).

**Fig 7:**
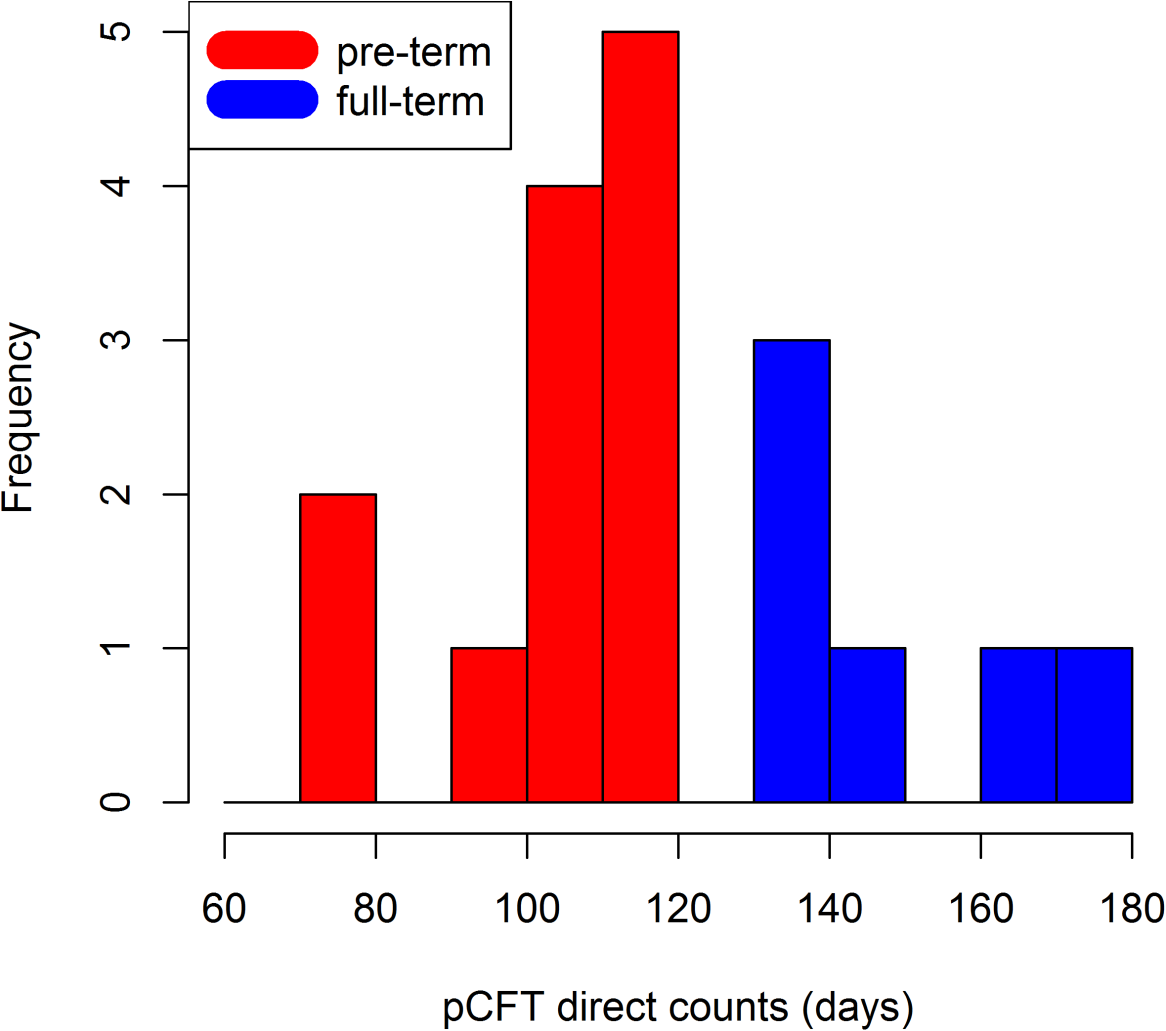
Histogram of the pCFT values in the Velia series.

Skinner and Dupras [70] investigated the location of the NL in primary teeth showing that it differs significantly among pre-term, full term and post term births. Similarly, it is possible to approximate the gestational age of Velia's individuals from their pCFTs. The current literature (reviewed in [27]) suggests that the deciduous central incisor crown starts mineralizing between 13 and 20 gestational weeks (91 and 140 days respectively). Assuming the average duration of a singleton pregnancy to be equivalent to 39 weeks (273 days), then the initial mineralization for the central deciduous incisors (expressed by the pCFT) in full term individuals would range between 182 and 133 days before birth. Therefore, it is reasonable to consider as pre-term individuals those that have a pCFT in the central incisors shorter than 133 days. The histogram of the pCFT in the Velia series is shown in Fig 7. Considering the shape of the sample distribution, showing a gap between 120 and 130 days, we can cautiously define a more conservative threshold between pre-term and full term individuals at 120 days of pCFT. Therefore, all the individuals having a pCFT equal or shorter than 120 days can be diagnosed as born pre-term. With this assumption in the Velia sample, we judge that 12 individuals were born pre-term and 6 full term. Among those identified as pre-term individuals, two (T.197 and T.243) survived long enough to form the NL.

## Discussion

The study of the portion of deciduous crowns that forms prenatally is of primary importance in understanding the timing and modality of fetal enamel development because it reflects life history parameters, such as the duration of pregnancy and stage of development at birth. It has the potential to open up a window on the mother’s health status during the last phases of pregnancy and also on any differences in the incidence of fetal stress events [18, 71].

In this context, the Velia sample provides a perfect opportunity to explore this potential further, in particular because of the exceptional preservation and visibility of the fine enamel microstructure. The data retrieved from the deciduous central incisors from Velia, i.e. the prenatal DSR, EER, CFT and the resulting regression equation, most likely represent the best reference so far available for any research aimed at estimating the prenatal CFT in ancient populations or in fossil specimens when prenatal incremental markings are indistinct. Moreover, for the first time with such resolution (but see [72] for a similar approach), it was possible to estimate the ratio between pre-term vs full term births in an archaeological population (Fig 7).

So far, studies on the prenatal portion of the tooth crown in deciduous teeth have mainly focused on reconstructing the ontogenetic trajectories of daily enamel secretion rates, DSR, and enamel extension rates, EER, with the aim of creating standards that can be applied to the human and primate fossil record [26, 27, 73]. In other circumstances also, for example, when poor preservation of the incremental features preclude the visualization of a complete temporal record of growth in prenatal enamel, thereby preventing a direct estimate of the pCFT, statistically based models of growth are still required to reconstruct enamel total formation times. Moreover, even when enamel and dentine microstructure can be imaged non-destructively (so-called virtual histology) [74–77] using techniques such as high resolution phase contrast synchrotron light microtomography or, in certain circumstances, with μMRI (magnetic resonance microimaging), a statistical model to evaluate tooth development is still required, especially when a fully readable virtual histological record cannot be retrieved.

To address these limitations, analytical approaches based on regression estimates of enamel growth parameters have been proposed and used to reconstruct tooth crown growth [25, 27, 48, 78].

However, to date, there is a need for more studies to collect additional data for deciduous enamel growth in past population that aim to assess whether differences exist in prenatal enamel growth then and now. This study adds to the data already available from archaeological horizons [24–26], and contributes to the challenge of not having to use modern reference series when studying archaeological skeletal collections.

The slope of the regression lines in Fig 1 suggests, in fact, that there is some stability across different populations. The regression slope of the Velia sample is only slightly different from that of Birch and Dean’s [27] original sample. In fact, the regression lines are sub-parallel and the main difference lies in the intercept value, suggesting that the DSR estimators are good proxies for these basic developmental processes in anatomically modern humans.

When applying the new regression formula to the Velia series, we observe a mean absolute difference between the direct count and the estimation of the pCFT of 11 days (range = -34 / 16 days). This level of error is probably acceptable in the context of an overall estimate of pCFT, because even with direct counts across the whole crown it would be difficult to achieve greater accuracy. Indeed, a number of factors can affect direct estimates based on counts of daily incremental markings, such as prism decussation, orientation and obliquity of the thin section [79], diagenesis and taphonomic history.

**Fig 8:**
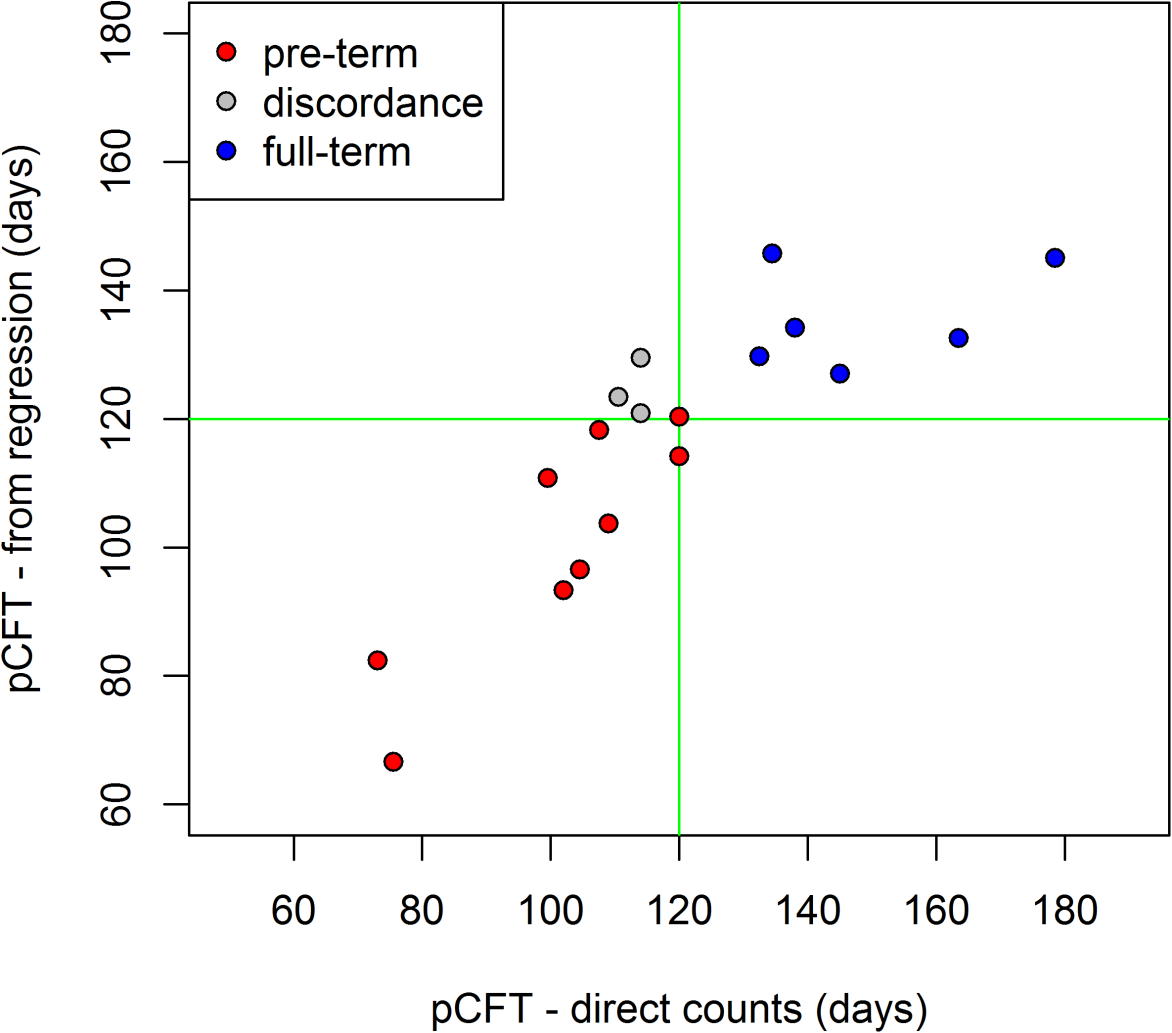
Scatterplot comparing the individual pCFT from direct counts and from the new regression formula. The thresholds at 120 days are marked as green lines. Gray dots represent the individuals in which the diagnosis is discordant.

A good agreement between the direct counts and the predictions from the regression formula is also achieved when estimating the pre-term vs full term birth ratio, as shown in Fig 8. The regression predicted values fail to identify as pre-term only three individuals, who are anyway close to the threshold of 120 days.

**Fig 9:**
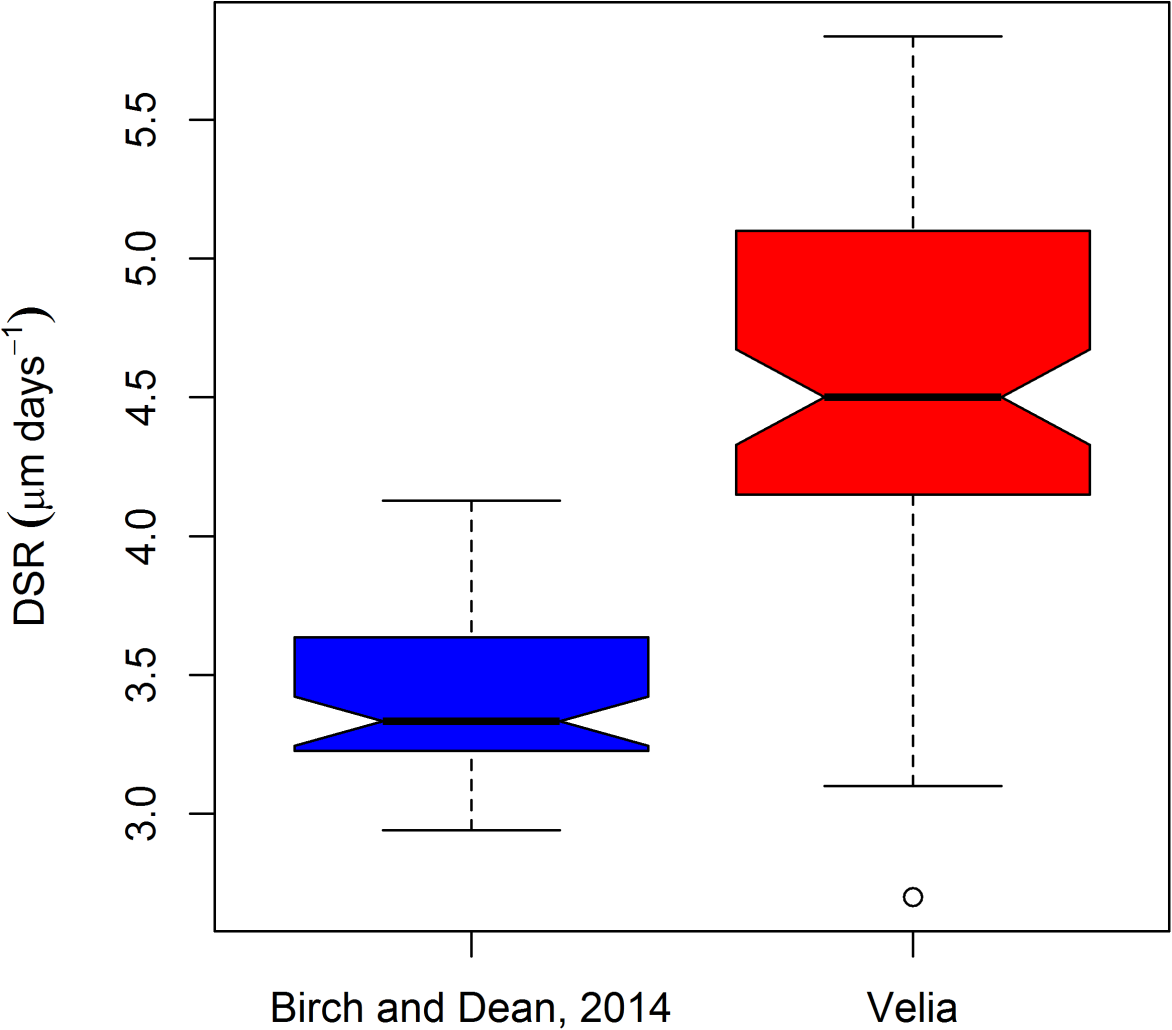
Boxplot of the DSR variability in the Velia series compared with the Birch and Dean [27] figures. The boxplot shows the median, the range (lower and upper whisker), the first quartile (lower hinge), and the third quartile (upper hinge). A single observation, exceeding the lower interquartile range times 1.5, is marked as an outlier.

The mean DSR for the Velia sample is faster than represented in the figures given in [27, 47]. In Fig 9 the boxplot of the the DSR derived from the single prism measurements (Velia n = 76, Birch and Dean [27] n = 54) is reported for both sets of data. The median DSR of the Velia incisors exceeded the values of the Birch [47] modern reference sample by more than a micrometer/day (Velia median DSR = 4.50, Birch [47] median DSR = 3.33).

Moreover, the DSR range of the Velia sample is greater: 2.7 to 5.8 μm day^-1^ as opposed to 2.94 to 4.13 μm day^-1^ reported in [47] for a modern series. The distributions of the DSR are statistically different when comparing the Velia sample and the modern series of Birch [47] (Wilcoxon rank sum test with continuity correction W = 3909, p< 0.001; Kolmogorov-Smirnov test D = 0.834, p< 0.001). The detail of the DSR statistic for all measured segments shows that there is a much larger variability in the Velia series (Fig 9). The lower limit of the Velia range results from the daily measurements along two prisms positioned very close to the cervical region in the T.258 individual (see S1 Table). Conversely, the higher median and the higher upper limits of the Velia distribution are harder to explain. It may simply be this discrepancy results from the extreme difference between the two samples. Alternatively, it may be the fact that the Velia measures are all confined to the prenatal enamel only (that forms such a large proportion of deciduous incisor enamel) and where the DSR may be higher and more constant due to the protected environment during fetal development. Indeed, a sharp deceleration in DSR has been observed immediately after birth in the deciduous first molar of a modern reference series [28, 48], followed by an acceleration, or catch-up phase, afterwards. Nonetheless, the DSR range observed in this study fits better with the range of 3.59 μm day^-1^ (inner enamel) to 5.15 μm day^-1^ (outer enamel) reported in [26] for a Medieval skeletal series in the central upper deciduous incisors.

Apart from considering the differences in the prenatal enamel formation rates between these samples, differences between pre- and post-natal enamel formation times need to be considered. Thus, as noted also by Mahoney [26], this mixing of two such different developmental periods is likely to influence the overall values of the DSRs reported.

The comparative literature about prenatal EER in human deciduous teeth is scanty. Histologically derived data on deciduous EER have been recently reported by Mahoney [26]. The mean initial EER value for the deciduous central incisor derived by Mahoney [26] doesn't differ significantly from the mean value obtained from the Velia series (Mahoney [26] mean initial EER = 52.03, N = 13, sd = 6.96; Velia mean EER in the cuspal-middle region = 53.53, N = 22, sd = 11.25; t test = 0.43, n = 33, p = 0.62). Moreover, Mahoney [26] reports a deceleration in the EER along the EDJ length which is fully comparable with the data of this contribution. As already noted in [33] for permanent teeth, there is a rather large variability in the trajectories of EERs, as proxied by the EDJ length (Fig 5), after crown initiation and on into the later phases of crown formation. What seems clear in all teeth studied is that after the first 50 days of pCFT, there is a substantial deceleration in the differentiation and recruitment of new ameloblasts along the EDJ.

The regression of the pCFT on the EDJ length derived from for the present paper dataset (Fig 4 and S2 Table) presents a mean error of the absolute residuals of 27 days (sd 21.38), higher than the one obtained from the regression of the direct cross-striation counts against the prism length for the same dataset. Therefore, even though it cannot be considered as such a good estimator of the pCFT, in conditions where the only measurable feature of the prenatal crown is the EDJ length, it can certainly be used as a rough indicator of the prenatal crown formation time.

Finally, when large numbers of measurements are possible across the whole enamel crown of several specimens, the evaluation of the topographical distribution of the DSR across the whole enamel section (depicted here in Fig 6B as a heat map) represents a promising tool for understanding the differences in the pattern of enamel secretion among human populations and fossil hominins. Further data collection will increase the predictive power of this technique and will help in evaluating the variability of the topographical distribution of the DSR in modern and fossil humans.

## Conclusions

The present contribution makes use of direct counts of enamel microstructural growth increments in order to estimate the prenatal DSR and EER of human deciduous central incisors in a pre-industrial population from the 1^st^ century CE Roman population of Velia. Our results on DSR and EER variation across the tooth crown fit perfectly with the evidence available in literature: the ameloblasts accelerate while moving from the EDJ toward the outer surface but slow down in their recruitment towards the most cervical formed enamel. Consequently, a new (with reference to the equations in [27]) regression formula of the prism length formed against the crown formation time is proposed for these deciduous central incisors, based on a robust statistical approach to analyze the data. This linear equation, with a single term that corresponds to the inverse of the generalized DSR, can be used for future studies aimed to quantify, in a simple way and following the method of Birch and Dean [27], the pCFT and the pre-term vs the full term birth ratio in studies of ancient populations. In the future, this methodology will be extended to include the whole deciduous dentition for the series from Velia. The goal is to establish broader reference standards for studies on the prenatal ontogenetic trajectories of crown formation that can then be compared with other archaeological and paleoanthropological specimens. Moreover, the comparison of the developmental trajectories among prenatal and postnatal enamel will contribute to the understanding of the different growth patterns, during fetal development and early infancy, both between taxa and among archaeological populations.

## Supporting information

S1 Table Individual morphological (skeletal and dental) age at death, presence/absence of the NL, pCFT (days) from thedirect count, pCFT (days) estimated by the regression of this paper, pCFT (days) estimated by the regression of Birch and Dean 2014, prenatal EDJ length (μm) in the buccal aspect, prenatal total EDJ length (μm), pCFT (days) estimated by the regression of Mahoney 2012. (PDF)

S2 Table Individual cross-striations counts, prism lengths and EDJ measurements. (PDF)

S1 Fig Histological sections along the central labiolingual plan of two deciduous central incisors from the necropolis of Velia. A: T. 312, perinatal individual without NL; B: T 98, 0–6 months showing the NL and ALs in the cervical portion of the crown.
(PDF)

## References

[1] Leeuwenhoek A. Microscopical observations of the structure of teeth and other bones. Phil Trans Royal Soc. 1665–1678; 12: 1002–1003

[2] Retzius A. Bemerkungen über den inneren Bau der Zähne, mitbesonderer Rücksicht auf dem in Zahnknochen vorkommenden Röhrenbau. Arch Anat Physiol. 1837; 486–566.

[3] Preiswerk G. Beitrage zur Kenntniss de Schmelzstrustur bei Säugethieren mit besonderer berucksichtigung der Ungulaten. Basel, Switzerland: Akadenische Buchhandlung; 1895.

[4] Pickerill HP. The Prevention of Dental Caries and Oral Sepsis. London: Baillibre, Tindall and Cox; 1912.

[5] von Asper H. Über die Braune Retzius’sche Parallelstreifung im Schmelz der menschlichen Zähne. Thesis (doctoral), Universität Zürich. 1916.

[6] Gysi A. Metabolism in adult enamel. Dental Digest. 1931; 37: 661–668.

[7] Gustafson G, Gustafson AG. Microanatomy and histochemistry of enamel In: Miles AEW editor. Structural and Chemical Organization of Teeth. London: Academic Press; 1967 pp. 135–162.

[8] Risnes S. Enamel apposition rate and the prism periodicity in human teeth. Scand J Dent Res. 1986; 94: 394–404. 3467418 10.1111/j.1600-0722.1986.tb01779.x

[9] Schour I, Massler M. Studies in tooth development: the growth pattern of human teeth, part 2. J Am Dent Ass. 1940; 27: 1918–1931.

[10] Kajiyama S. Total number of regular incremental lines in the enamel of human permanent teeth. Nihon U Dent J. 1965; 39: 77–83.

[11] Takiguchi H. Chronologic relationship of human tooth crown formation. Nihon Univ Dent J. 1966; 40: 391–397.

[12] Schour I. Neonatal line in enamel and dentin of human deciduous teeth and first permanent molar. J Am Dent Assoc. 1936; 23: 1946–1955. 10.1177/00220345460250030601 20988348

[13] Boyde A. Estimation of age at death of young human skeletal remains from incremental lines in dental enamel. Excerpta Med Int Congr Series. 1963; 80: 36–46.

[14] Boyde A. The structure and development of mammalian enamel. Ph.D. Thesis, University of London. 1964.

[15] Dean MC. Growth layers and incremental markings in hard tissues; a review of the literature and some preliminary observations about enamel structure in *Paranthropus boisei*. J Hum Evol 1987; 16: 157–172.

[16] FitzGerald CM. Do enamel microstructures have regular time dependency? Conclusions from the literature and a large-scale study. J Hum Evol. 1998; 35:371–386. doi: 10.1006/jhev.1998.0232 9774500 10.1006/jhev.1998.0232

[17] Smith TM. Experimental determination of the periodicity of incremental features in enamel. J Anat. 2006; 208:99–114. doi: 10.1111/j.1469-7580.2006.00499.x 16420383 10.1111/j.1469-7580.2006.00499.x PMC2100182

[18] Hillson S.Tooth Development in Human Evolution and Bioarchaeology. Cambridge, UK: Cambridge University Press; 2014.

[19] Lacruz RS, Hacia JG, Bromage TG, Boyde A., Lei Y, Xu Y, et al The circadian clock modulates enamel development. J Biol Rhythms. 2012; 27: 237–245. doi: 10.1177/0748730412442830 22653892 10.1177/0748730412442830 PMC3511783

[20] Zheng L, Seon YJ, Mourao MA, Schnell S, Kim D, Harada H, et al Circadian rhythms regulate amelogenesis. Bone. 2013; 55: 158–165. doi: 10.1016/j.bone.2013.02.011 23486183 10.1016/j.bone.2013.02.011 PMC3650122

[21] Zheng L, Ehardt L, McAlpin B, About I, Kim D, Papagerakis., et al The tick tock of odontogenesis. Exp. Cell Res. 2014; 325: 83–89. doi: 10.1016/j.yexcr.2014.02.007 24582863 10.1016/j.yexcr.2014.02.007 PMC4072747

[22] Lacruz RS. Genetic regulation of amelogenesis and implications for hominin ancestors In: Boughner J.C., Rolian C editors. Developmental Approaches to Human Evolution. New York: John Wiley and Sons; 2016 pp.61–75.

[23] Simpson SW. Reconstructing patterns of growth disruption from enamel microstructure In: Hoppa RD, FitzGerald CM, editors. Human growth in the past. Cambridge, UK: Cambridge University Press; 1999 pp. 241–263.

[24] Mahoney P. Human deciduous mandibular molar incremental enamel development. Am J Phys Anthropol. 2011; 144:204–214. doi: 10.1002/ajpa.21386 20740658 10.1002/ajpa.21386

[25] Mahoney P. Incremental enamel development in modern human deciduous anterior teeth. Am J Phys Anthropol. 2012; 147: 637–651. doi: 10.1002/ajpa.22029 22331636 10.1002/ajpa.22029

[26] Mahoney P. Dental fast track: prenatal enamel growth, incisor eruption and weaning in human infants. Am J Phys Anthropol. 2015; 156: 407–421. doi: 10.1002/ajpa.22666 25388809 10.1002/ajpa.22666

[27] Birch W, Dean MC. A method of calculating human deciduous crown formation times and of estimating the chronological ages of stressful events occurring during deciduous enamel formation. J Forensic Leg Med. 2014; 22: 127–144. doi: 10.1016/j.jflm.2013.12.002 24485438 10.1016/j.jflm.2013.12.002

[28] Macchiarelli R, Bondioli L, Debénath A, Mazurier A, Tournepiche JF, Birch W, et al How Neanderthal molar teeth grew. Nature. 2006; 444: 748–751. doi: 10.1038/nature05314 17122777 10.1038/nature05314

[29] Huda T, Bowman JE. Variation in cross-striation number between striae in an archaeological population. Int J Osteoarchaeol. 1994; 4:49–52.

[30] FitzGerald CM, Hillson S. Deciduous tooth growth in an ancient Greek infant cemetery In: Koppe T, Meyer G, Alt K, editors. Comparative dental morphology. Front Oral Biol Basel: Karger 2009 pp. 178–183. 10.1159/000242414 19828993

[31] Shellis RP. Variations in growth of the enamel crown in human teeth and a possible relationship between growth and enamel structure. Arch Oral Biol. 1984; 29: 671–682. 10.1016/0003-9969(84)90175-4 6594102

[32] Dean MC. Extension rates and growth in tooth height of modern human and fossil hominin canines and molars In: Koppe T, Meyer G, Alt K, editors. Comparative dental morphology. Front Oral Biol Basel: Karger 2009 pp. 68–73. 10.1159/000242394 19828973

[33] Guatelli Steinberg D, Bruce A, Floyd BA, Dean MC, Reid D. Enamel extension rate patterns in modern human teeth: Two approaches designed to establish an integrated comparative context for fossil primates. J Hum Evol. 2012; 63: 475–486. doi: 10.1016/j.jhevol.2012.05.006 22748383 10.1016/j.jhevol.2012.05.006

[34] Smith P, Avishai G. The use of dental criteria for estimating postnatal survival in skeletal remains of infants. J Archaeol Sc. 2005; 32: 83–89.

[35] Katzenberg MA, Oetelaar G, Oetelaar J, FitzGerald CM, Yang D, Saunders SR. Identification of historical human skeletal remains: a case study using skeletal and dental age, history and DNA. Int J Osteoarchaeol. 2005; 15:61–72.

[36] Skinner MF, Anderson GS. Individualization and enamel histology: a case report in forensic anthropology. J Forensic Sc. 1991; 36:939–948. 1856657

[37] Antoine D, Hillson S, Dean MC. The developmental clock of dental enamel: a test for the periodicity of prism cross-striations in modern humans and an evaluation of the most likely sources of error in histological studies of this kind. J Anat. 2009; 214: 45–55. doi: 10.1111/j.1469-7580.2008.01010.x 19166472 10.1111/j.1469-7580.2008.01010.x PMC2667916

[38] Witzel C. Echoes from birth—mutual benefits for physical and forensic anthropology by applying increment counts in enamel of deciduous teeth for aging. Anthropol Anz. 2014; 71(1–2): 87–103. 24818441 10.1127/0003-5548/2014/0386

[39] Smith T M, Reid D G, Olejniczkac A J, Bailey S, Glanz M, Viola B et al, Dental development and age at death of Middle Paleolithic juvenile hominin from Obi-Rakhmat Grotto, Uzbekistan In Condemi S, Weniger GC editors. Continuity and discontinuity in the peopling of Europe: one hundred fifty years of Neanderthal study, Vertebrate Paleobiology and Paleoanthropology. London-New York: Springer Science + Business Media; 2011 pp. 155–163.

[40] Stringer CB, Dean MC, Martin RD. A comparative study of cranial and dental development within a recent British sample and among Neandertals In: De Rousseau CJ editor. Primate Life History and Evolution. New York: Wiley-Liss; 1990 pp 115–152.

[41] Dean MC. Tooth microstructure tracks the pace of human life-history evolution. Proc Biol Sci. 2006; 273(1603): 2799–2808. doi: 10.1098/rspb.2006.3583 17015331 10.1098/rspb.2006.3583 PMC1664636

[42] Witzel C, Kierdorf U, Schultz M, Kierdorf H. Insights from the inside—Histological Analysis of Abnormal Enamel Microstructure Associated with Hypoplastic Enamel Defects in Human Teeth. Am J Phys Anthropol. 2008; 136: 400–414 doi: 10.1002/ajpa.20822 18350581 10.1002/ajpa.20822

[43] Sabel N, Johansson C, Kühnisch J, Robertson A, Steiniger F, Norén JG, et al Neonatal lines in the enamel of primary teeth—A morphological and scanning electron microscopic investigation. Arch Oral Biol. 2008; 53(10): 954–963. doi: 10.1016/j.archoralbio.2008.05.003 18589400 10.1016/j.archoralbio.2008.05.003

[44] Zanolli C, Bondioli L, Manni F, Rossi P, Macchiarelli R. Gestation length, mode of delivery, and neonatal line-thickness variation. Hum Biol. 2011; 83(6): 695–713. doi: 10.3378/027.083.0603 22276969 10.3378/027.083.0603

[45] Seow KW, Young WG, Tsang AKL. A study of primary dental enamel from preterm and full-term children using light and scanning electron microscopy. Pediatr Dent. 2005; 27(5): 374–379. 16435636

[46] Dean MC. 2D or not 2D, and other interesting questions about enamel: reply to Macho et al. (2003). J Hum Evol. 2004; 46: 633–640. doi: 10.1016/j.jhevol.2004.03.001 15120270 10.1016/j.jhevol.2004.03.001

[47] Birch W. Incremental growth of deciduous tooth enamel. PhD Thesis, University College London. 2011.

[48] Birch W, Dean MC. Rates of enamel formation in human deciduous teeth. In: Koppe T, Meyer G, Alt KW, editors. Comparative Dental Morphology. Front Oral Biol. 2009; 13: 116–120. doi: 10.1159/000242402 Epub 2009 Sep 21. 19828981 10.1159/000242402

[49] Norén JG. Enamel structure in deciduous teeth in low-birth weight infants. Acta Odontol Scand. 1983; 41: 335–362. 10.3109/00016358309162347 6581675

[50] Rythén M, Norén JG, Sabel N, Steiniger F, Niklasson A, Hellström A. Morphological aspects of dental hard tissues in primary teeth from preterm infants. Int J Paediatr Dent. 2008; 18: 397–406. doi: 10.1111/j.1365-263X.2008.00928.x 18637047 10.1111/j.1365-263X.2008.00928.x

[51] Craig O, Biazzo M, O'Connell TC, Garnsey P, Martinez-Labarga C, Lelli R, et al Stable isotopic evidence for diet at the Imperial Roman coastal site of Velia (1st and 2nd centuries AD) in Southern Italy. Am J Phys Anthropol. 2009; 139(4): 572–583. doi: 10.1002/ajpa.21021 19280672 10.1002/ajpa.21021

[52] Bondioli L, Nava A, Rossi PF, Sperduti A. Diet and health in Central-Southern Italy during the Roman Imperial Time. Acta Imeko. 2016; 5(2): 19–25.

[53] Janardhanan M, Umadethan B, Biniraj KR, Kumar VRB, Rakesh S. Neonatal line as a linear evidence of live birth: Estimation of postnatal survival of a new born from primary tooth germs. J Forensic Dent Sci. 2011; 3(1): 8–13. doi: 10.4103/0975-1475.85284 22022132 10.4103/0975-1475.85284 PMC3190441

[54] Fiammenghi CA. La Necropoli di Elea-Velia: qualche osservazione preliminare In: Elea-Velia. Le Nuove ricerche. Quaderni del Centro Studi Magna Grecia 1. Pozzuoli: Naus Editoria; 2003 pp. 49–61.

[55] Caropreso S, Bondioli L, Capannolo D, Cerroni L, Macchiarelli R, Condò SG. Thin sections for hard tissues histology: a new procedure. J Microsc. 2000; 199: 244–247. 10971805 10.1046/j.1365-2818.2000.00731.x

[56] Scheuer L, Black S. Developmental Juvenile Osteology. Amsterdam: Elsevier Academic Press; 2000.

[57] AlQahtani SJ, Hector MP, Liversidge HM. Brief communication: The London atlas of human tooth development and eruption. Am J Phys Anthropol. 2010; 142: 481–490 doi: 10.1002/ajpa.21258 20310064 10.1002/ajpa.21258

[58] Ubelaker DH. Human skeletal remains: excavation, analysis, interpretation. Chicago: Aldine Publishing Co. Inc.; 1978.

[59] Fazekas IG, Kosa F. Forensic Fetal Osteology. Budapest: Akademiai Kiado; 1978.

[60] Maresh MM. Measurements from Roentgenograms In: McCammon RW, editor. Human Growth and Development. Springfield, IL: C. C. Thomas; 1970 pp 157–200.

[61] Dean MC. A histological method that can be used to estimate the time taken to form the crown of a permanent tooth In: Bell LS, editor. Forensic microscopy for skeletal tissues: methods and protocols. Method in Molecular Biology vol. 915, Springer Science + Business Media; 2012 pp.89–100. 10.1007/978-1-61779-977-8_5 22907403

[62] Lewis SJ. Quantifying measurement error. In: Anderson S editor. Current and recent research in osteoarchaeology 2: Proceedings of the 4th, 5th and 6th meetings of the Osteoarchaeological Research Group. Oxford: Oxbow Books; 1999. pp. 54–55.

[63] Koller M, Stahel WA. Sharpening Wald-type inference in robust regression for small samples. Comput Stat Data Anal. 2011; 55(8): 2504–2515.

[64] Wood SN. Generalized Additive Models: an Introduction with R. Boca Raton: Chapman and Hall-CRC; 2006.

[65] Cleveland WS, Grosse E, Shyu WM. Local regression models in S In: Chambers JM, Hastie TJ editors. Statistical Models in S. New York: Chapman & Hall; 1993 pp. 309–376

[66] R core team. R: A language and environment for statistical computing. R Foundation for Statistical Computing, Vienna, Austria 2017 URL https://www.R-project.org/.

[67] Rousseeuw P, Croux C, Todorov V, Ruckstuhl A, Salibian-Barrera M, et al. Robustbase: Basic Robust Statistics. R package version 0.92–6. 2016. URL http://CRAN.R-project.org/package=robustbase.

[68] Boyde A. Developmental interpretations of dental microstructure In: De Rousseau CJ, editor. Primate Life History and Evolution. Monographs in Primatology Vol 14 New-York: Wiley Liss; 1990 pp. 229–267.

[69] Zanolli C, Dean C, Rook L, Bondioli L, Mazurier A, Macchiarelli R. Enamel thickness and enamel growth in Oreopithecus: Combining microtomographic and histological evidence. C R Palevol. 2016; 15: 209–229.

[70] Skinner M, Dupras T. Variation in birth timing and location of the neonatal line in human enamel. J Forensic Sci. 1993; 38(6):1383–1390. 8263481

[71] Guatelli Steinberg D. What Teeth Reveal about Human Evolution. Cambridge UK: Cambridge University Press; 2016.

[72] Schwartz JH, Houghton F, Macchiarelli R, Bondioli L. Skeletal Remains from Punic Carthage Do Not Support Systematical Sacrifice of Infants. Plos One. 2010; 5(2):e9177 doi: 10.1371/journal.pone.0009177 20174667 10.1371/journal.pone.0009177 PMC2822869

[73] Smith TM, Tafforeau P, Le Cabec A, Bonnin A, Houssaye A, Pouech J, et al Dental ontogeny in Pliocene and Early Pleistocene hominins. PLoS One. 2015; 10:e0118118 doi: 10.1371/journal.pone.0118118 25692765 10.1371/journal.pone.0118118 PMC4334485

[74] Zanolli C, Bondioli L, Coppa A, Dean CM, Bayle P, Candilio F, et al The late Early Pleistocene human dental remains from Uadi Aalad and Mulhuli-Amo (Buia), Eritrean Danakil: macromorphology and microstructure. J Hum Evol. 2014; 74: 96–113. doi: 10.1016/j.jhevol.2014.04.005 24852385 10.1016/j.jhevol.2014.04.005

[75] Le Cabec A, Tang N, Tafforeau P. Accessing developmental information of fossil hominin teeth using new synchrotron microtomography- based visualization techniques of dental surfaces and interfaces. PLoS One. 2015; 10:e0123019 doi: 10.1371/journal.pone.0123019 25901602 10.1371/journal.pone.0123019 PMC4406681

[76] Tafforeau P, Smith TM. Nondestructive imaging of hominoid dental microstructure using phase contrast X-ray synchrotron microtomography. J Hum Evol. 2008; 54: 272–278. doi: 10.1016/j.jhevol.2007.09.018 18045654 10.1016/j.jhevol.2007.09.018

[77] Tafforeau P, Zermeno JP, Smith TM. Tracking cellular-level enamel growth and structure in 4D with synchrotron imaging. J Hum Evol. 2012; 62: 424–428. doi: 10.1016/j.jhevol.2012.01.001 22304852 10.1016/j.jhevol.2012.01.001

[78] Dean MC. Retrieving chronological age from dental remains of early fossil hominins to reconstruct human growth in the past. Phil Trans R Soc B. 2010; 365: 3397–3410. doi: 10.1098/rstb.2010.0052 20855313 10.1098/rstb.2010.0052 PMC2981956

[79] Smith TM, Reid DJ, Sirianni JE. The accuracy of histological assessment of dental development and age at death. J Anat. 2006; 208: 125–138. doi: 10.1111/j.1469-7580.2006.00500.x 16420385 10.1111/j.1469-7580.2006.00500.x PMC2100178

