# Supplementary material for: New regression formula to estimate the prenatal crown formation time of human deciduous central incisors derived from a Roman Imperial sample (Velia, Salerno, Italy, I-II cent. CE)
Source: PLoS One. 2017 Jul 12;12(7):e0180104. doi: 10.1371/journal.pone.0180104 (PMC5507505; doi:10.1371/journal.pone.0180104)
Supplement: S1 Table — (PDF) [file pone.0180104.s001.pdf]

New Regression Formula to Estimate the Prenatal Crown Formation Time of Human Deciduous Central Incisors Derived from a Roman Imperial Sample (Velia, Salerno, Italy, I-II cent. CE)

Alessia Nava, Luca Bondioli, Alfredo Coppa, Christopher Dean, Paola Francesca Rossi and Clément Zanolli

Supplementary information table 1

**Individual**	**Morphological (skeletal and dental) age at death**	**NL present**	**pCFT (days) direct count**	**pCFT (days) estimated by the new regression**	**pCFT (days) Birch and Dean 2014**	**Prenatal buccal EDJ length (µm)**	**Prenatal total EDJ length (µm)**	**pCFT (days) Mahoney 2012**
Velia T98	0-6 months	Y	179	145	187	4912	10517	224
Velia T142	0-6 months	N	105	97	131	3244	7650	98
Velia T155 I	perinatal	N	145	127	173	3628	8643	142
Velia T168 I	0-6 months	N	120	120	159	4949	9886	196
Velia T197	0-3 months	Y	120	114	152	5227	10537	225
Velia T221	perinatal	N	76	67	97	3240	6909	66
Velia T229	5-7 months	Y	138	134	175	5018	10305	215
Velia T237	perinatal	N	102	93	134	4072	7788	104
Velia T243	perinatal	Y	100	111	154	5344	10174	209
Velia T252	perinatal	N	164	133	206	5218	11178	253
Velia T301	0-3 months	N	109	104	153	4063	9617	184
Velia T312	0-3 months	N	133	130	190	5766	12496	310
Velia T330	perinatal	N	108	118	170	4183	10264	213
Velia T344	perinatal	N	114	130	183	4889	11218	255
Velia T349	perinatal	N	135	146	201	3552	7430	89
Velia T399	0-3 months	Y	114	121	166	4055	9172	165
Velia T422	2-3 months	N	73	82	115	2481	7181	78
Velia T438	perinatal	N	111	123	176	4680	9477	178
