# Supplementary material for: New regression formula to estimate the prenatal crown formation time of human deciduous central incisors derived from a Roman Imperial sample (Velia, Salerno, Italy, I-II cent. CE)
Source: PLoS One. 2017 Jul 12;12(7):e0180104. doi: 10.1371/journal.pone.0180104 (PMC5507505; doi:10.1371/journal.pone.0180104)
Supplement: S2 Table — (PDF) [file pone.0180104.s002.pdf]

New Regression Formula to Estimate the Prenatal Crown Formation Time of Human Deciduous Central Incisors Derived from a Roman Imperial Sample (Velia, Salerno, Italy, I-II cent. CE)

Alessia Nava, Luca Bondioli, Alfredo Coppa, Christopher Dean, Paola Francesca Rossi and Clément Zanolli

Supplementary information table 2

Individual	Point	Prism Length (µm)	Number of cross-striation	Length on the EDJ (µm)	DSR	EER	Dental arch	NL present
Velia T98	Z1	160.2	40.0	2159	4.0	54.0	superior	Y
Velia T98	Z2	275.3	76.5	1990	3.6	26.0	superior	Y
Velia T98	Z3	237.5	62.0	763	3.8	12.3	superior	Y
Velia T142	Z1	187.0	42.0	2435	4.5	58.0	inferior	N
Velia T142	Z2	128.8	29.5	809	4.4	27.4	inferior	N
Velia T142	Z3	132.3	33.0	-	4.0	-	inferior	N
Velia T155 I	Z1	217.9	59.0	2222	3.7	37.7	superior	N
Velia T155 I	Z2	140.8	28.0	760	5.0	27.1	superior	N
Velia T155 I	Z3	123.7	32.0	646	3.9	20.2	superior	N
Velia T155 I	Z4	107.1	26.0	-	4.1	-	superior	N
Velia T168 I	Z1	277.2	61.5	3407	4.5	55.4	superior	N
Velia T168 I	Z2	159.8	35.0	1032	4.6	29.5	superior	N
Velia T168 I	Z3	121.5	23.5	510	5.2	21.7	superior	N
Velia T197	Z1	323.3	69.5	3608	4.7	51.9	superior	Y
Velia T197	Z2	124.0	29.0	1014	4.3	35.0	superior	Y
Velia T197	Z3	82.5	21.5	605	3.8	28.2	superior	Y
Velia T221	Z1	158.4	41.0	2205	3.9	53.8	inferior	N
Velia T221	Z2	92.2	21.0	687	4.4	32.7	inferior	N
Velia T221	Z3	58.6	13.5	348	4.3	25.8	inferior	N
Velia T229	Z1	259.5	61.0	3322	4.3	54.5	superior	Y
Velia T229	Z2	149.3	31.0	761	4.8	24.5	superior	Y
Velia T229	Z3	213.8	46.0	935	4.6	20.3	superior	Y
Velia T237	Z1	198.9	45.5	2470	4.4	54.3	inferior	N
Velia T237	Z2	110.9	25.5	961	4.3	37.7	inferior	N
Velia T237	Z3	79.4	19.0	641	4.2	33.7	inferior	N
Velia T237	Z4	43.9	12.0	-	3.7	-	inferior	N
Velia T243	Z1	197.7	39.0	3288	5.1	84.3	superior	Y
Velia T243	Z2	89.6	17.5	654	5.1	37.4	superior	Y
Velia T243	Z3	119.5	22.0	858	5.4	39.0	superior	Y
Velia T243	Z4	107.1	21.0	544	5.1	25.9	superior	Y
Velia T252	Z1	94.5	23.5	1389	4.0	59.1	superior	N
Velia T252	Z2	141.2	36.0	1428	3.9	39.7	superior	N
Velia T252	Z3	99.4	24.5	847	4.1	34.6	superior	N
Velia T252	Z4	91.5	22.0	468	4.2	21.3	superior	N
Velia T252	Z5	81.8	23.5	501	3.5	21.3	superior	N

Velia T252	Z6	47.8	13.0	285	3.7	22.0	superior	N
Velia T252	Z7	37.2	14.0	300	2.7	21.4	superior	N
Velia T252	Z8	21.6	7.0	-	3.1	-	superior	N
Velia T301	Z1	74.7	18.0	740	4.2	41.1	inferior	N
Velia T301	Z2	124.3	30.0	1643	4.1	54.8	inferior	N
Velia T301	Z3	59.5	14.0	763	4.2	54.5	inferior	N
Velia T301	Z4	74.8	17.0	917	4.4	53.9	inferior	N
Velia T301	Z5	148.0	30.0	-	4.9	-	inferior	N
Velia T312	Z1	127.8	28.5	1805	4.5	63.3	superior	N
Velia T312	Z2	83.2	19.0	821	4.4	43.2	superior	N
Velia T312	Z3	88.1	20.0	879	4.4	43.9	superior	N
Velia T312	Z4	124.4	27.0	1011	4.6	37.5	superior	N
Velia T312	Z5	81.6	19.0	490	4.3	25.8	superior	N
Velia T312	Z6	96.6	19.0	760	5.1	40.0	superior	N
Velia T330	Z1	167.4	33.0	1821	5.1	55.2	superior	N
Velia T330	Z2	157.9	29.0	1546	5.4	53.3	superior	N
Velia T330	Z3	77.1	16.0	444	4.8	27.7	superior	N
Velia T330	Z4	58.7	12.5	372	4.7	29.8	superior	N
Velia T330	Z5	87.7	17.0	-	5.2	-	superior	N
Velia T344	Z1	135.4	28.5	1690	4.8	59.3	superior	N
Velia T344	Z2	184.9	33.5	1910	5.5	57.0	superior	N
Velia T344	Z3	62.4	12.0	428	5.2	35.7	superior	N
Velia T344	Z4	142.3	27.0	861	5.3	31.9	superior	N
Velia T344	Z5	75.8	13.0	-	5.8	-	superior	N
Velia T349	Z1	105.0	23.5	1584	4.5	67.4	inferior	N
Velia T349	Z2	211.3	40.5	1048	5.2	25.9	inferior	N
Velia T349	Z3	155.9	30.5	920	5.1	30.2	inferior	N
Velia T349	Z4	108.4	22.0	-	4.9	-	inferior	N
Velia T349	Z5	95.5	18.0	-	5.3	-	inferior	N
Velia T399	Z1	134.9	30.5	1209	4.4	39.7	superior	N
Velia T399	Z2	160.3	28.5	1139	5.6	40.0	superior	N
Velia T399	Z3	124.2	24.0	837	5.2	34.9	superior	N
Velia T399	Z4	141.3	31.0	870	4.6	28.1	superior	N
Velia T422	Z1	144.2	27.0	1608	5.3	59.6	superior	N
Velia T422	Z2	132.7	23.5	873	5.6	37.1	superior	N
Velia T422	Z3	105.4	22.5	-	4.7	-	superior	N
Velia T438	Z1	198.9	37.0	2419	5.4	65.4	superior	N
Velia T438	Z2	130.5	26.5	1443	4.9	54.5	superior	N
Velia T438	Z3	40.0	10.0	387	4.0	38.7	superior	N
Velia T438	Z4	44.5	9.0	431	4.9	47.9	superior	N
Velia T438	Z5	158.6	28.0	-	5.7	-	superior	N
