# Supplementary figures and images for: New regression formula to estimate the prenatal crown formation time of human deciduous central incisors derived from a Roman Imperial sample (Velia, Salerno, Italy, I-II cent. CE)
Source: PLoS One. 2017 Jul 12;12(7):e0180104. doi: 10.1371/journal.pone.0180104 (PMC5507505; doi:10.1371/journal.pone.0180104)

500  $\mu\text{m}$

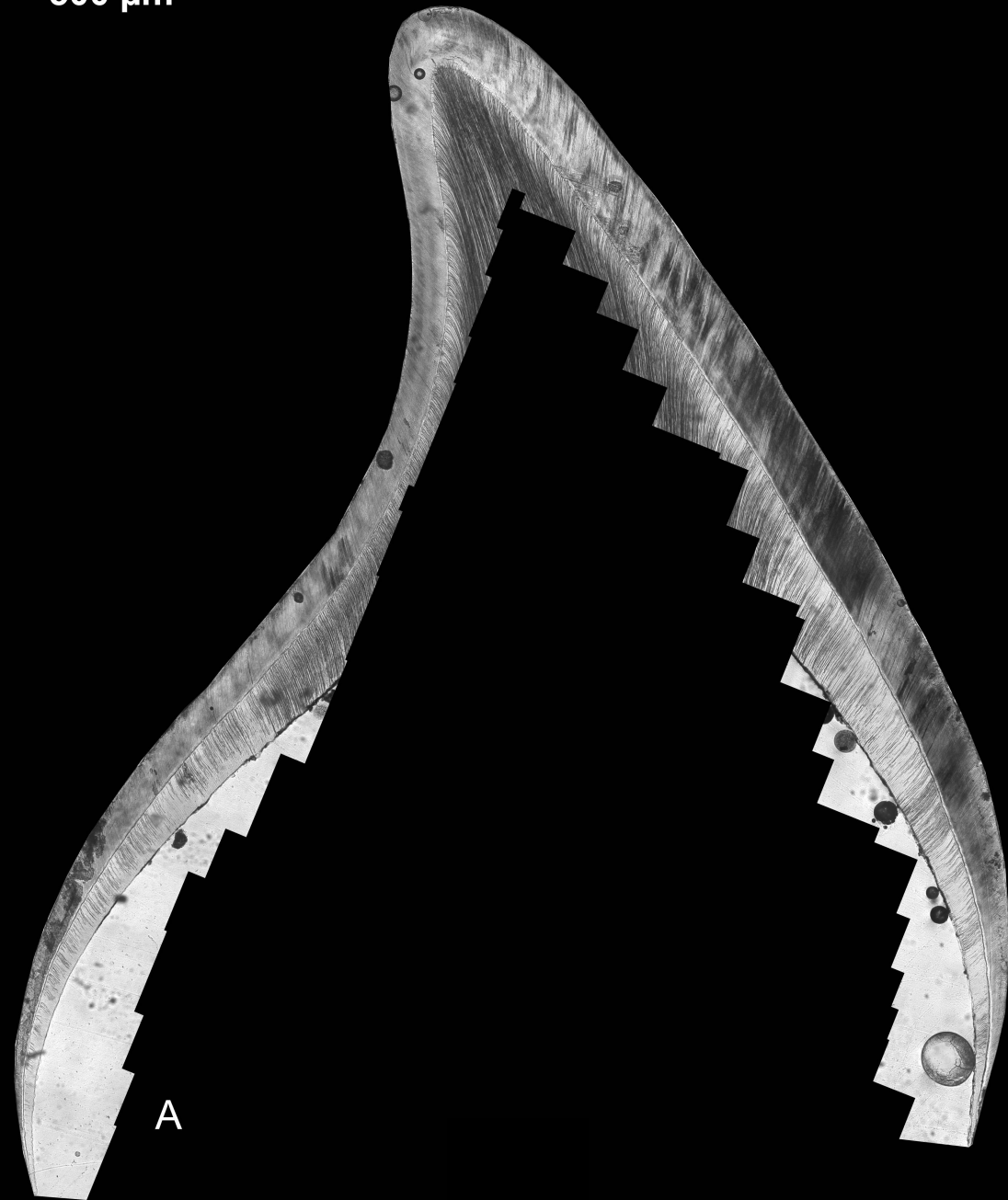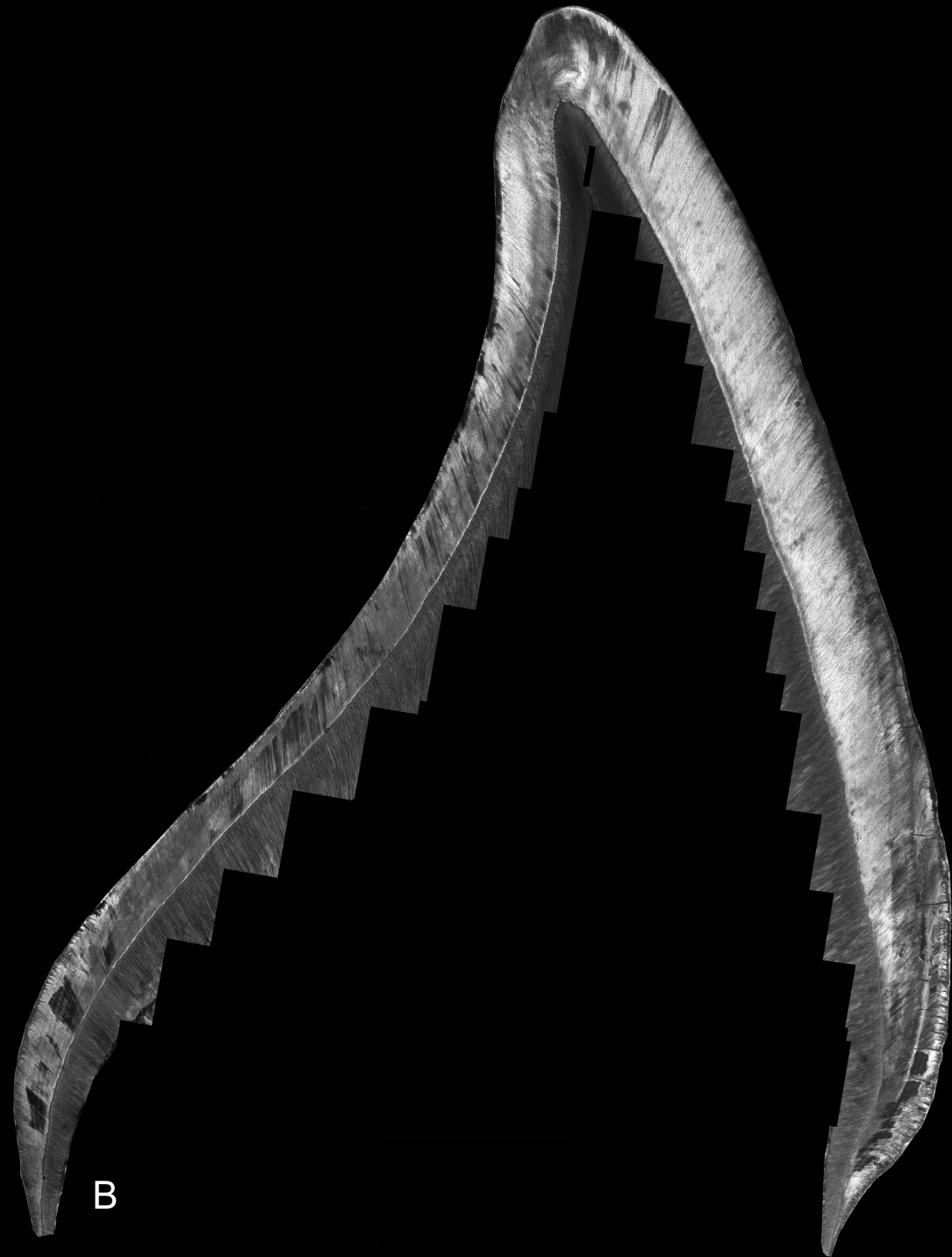

Supplement: S1 Fig — A: T. 312, perinatal individual without NL; B: T 98, 0–6 months showing the NL and ALs in the cervical portion of the crown. (PDF) [file pone.0180104.s003.pdf]
